# Supplementary figures and images for: Gamma oscillations in primate primary visual cortex are severely attenuated by small stimulus discontinuities
Source: PLoS Biol. 2022 Jun 14;20(6):e3001666. doi: 10.1371/journal.pbio.3001666 (PMC9197048; doi:10.1371/journal.pbio.3001666)

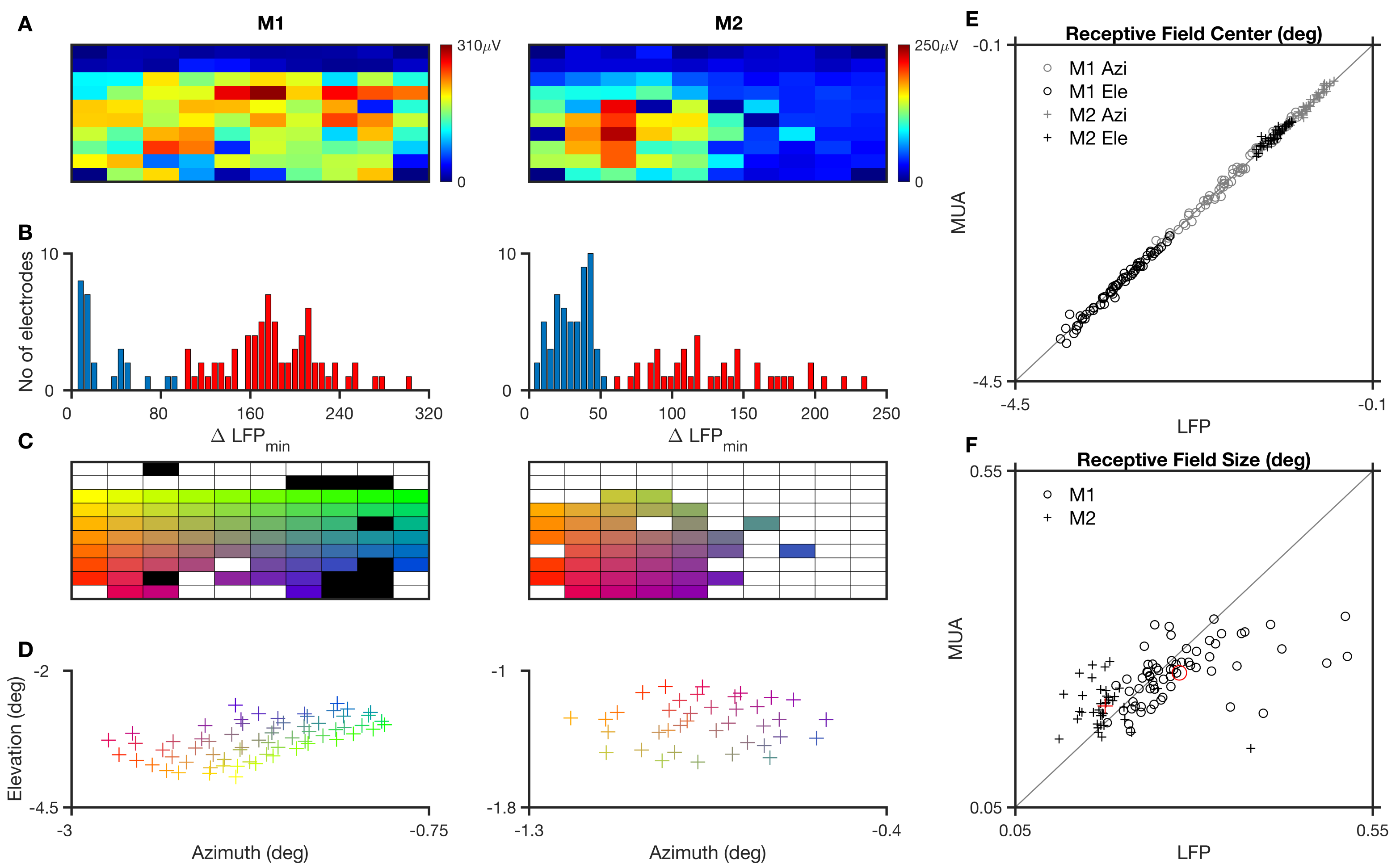

Supplement: S1 Fig — (A) Magnitude of the evoked response produced by mapping stimuli (for details, see Materials and methods) is shown across the grid of electrodes, averaged across RF mapping sessions (7 sessions each in M1 and M2). (B) Electrodes showing a response above an arbitrary threshold (100 for M1, 60 for M2) are chosen as consistently good electrodes (indicated in red) for further analyses. (C) A color-coded schematic of the physical microelectrode grid and (D) the corresponding RF centers of these sites in the visual space determined as per the mapping algorithm described in the Materials and methods. Mapping based on firing rates (MUA) was done similarly by using the change in mean firing rate from baseline to stimulus period as the “response” metric. (E) Scatter of the estimated azimuth and elevation of RF centers for the selected electrodes. The RF center estimated from LFP and MUA were closely matched in both monkeys. (F) Scatter of RF size across the selected electrodes. Mean sizes are shown in red. Figure data are located at https://doi.org/10.5281/zenodo.6523772. LFP, local field potential; M1, Monkey 1; M2, Monkey 2; MUA, multiunit activity; RF, receptive field. (PDF) [file pbio.3001666.s001.pdf]

**A**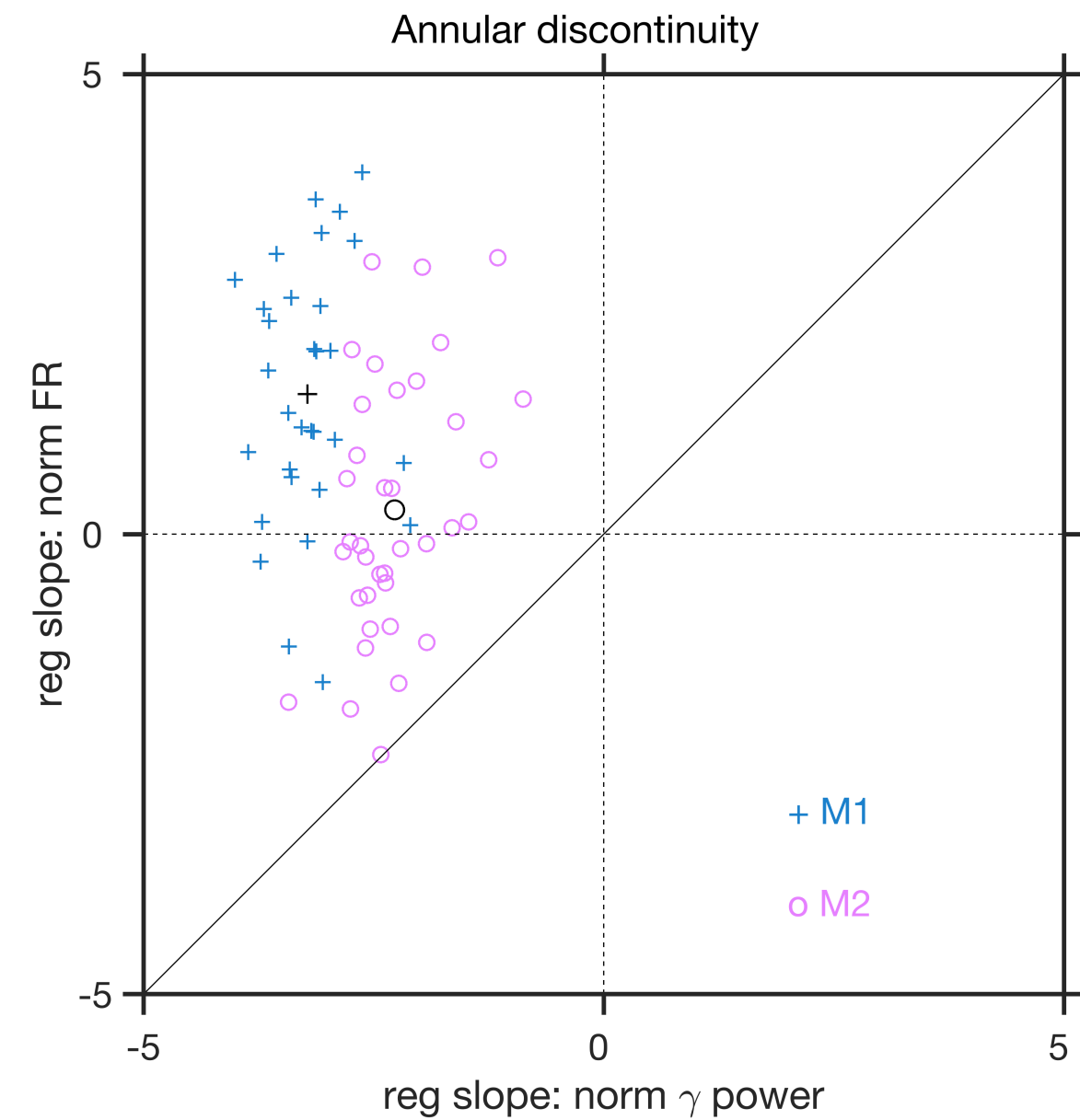**B**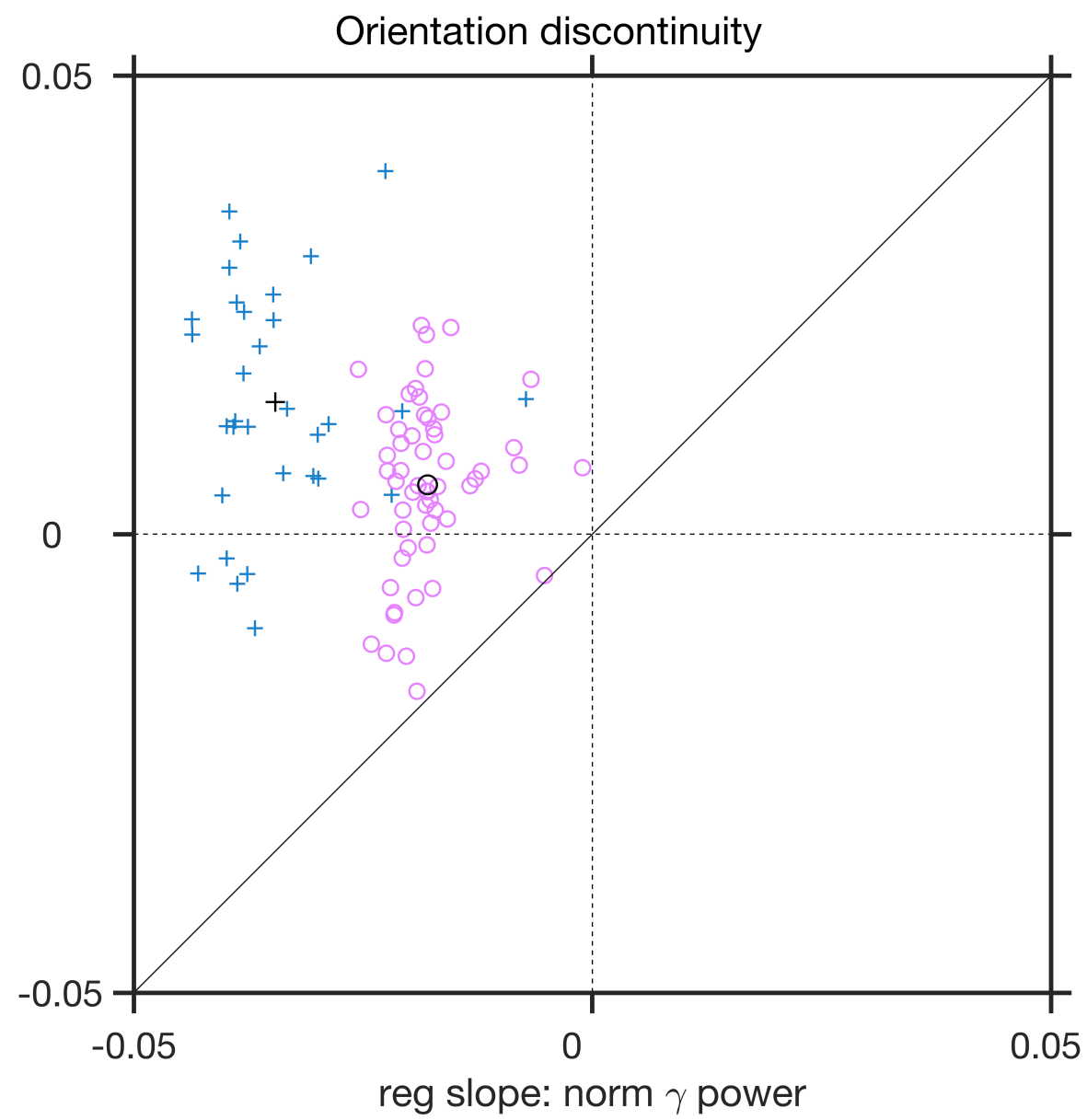**C**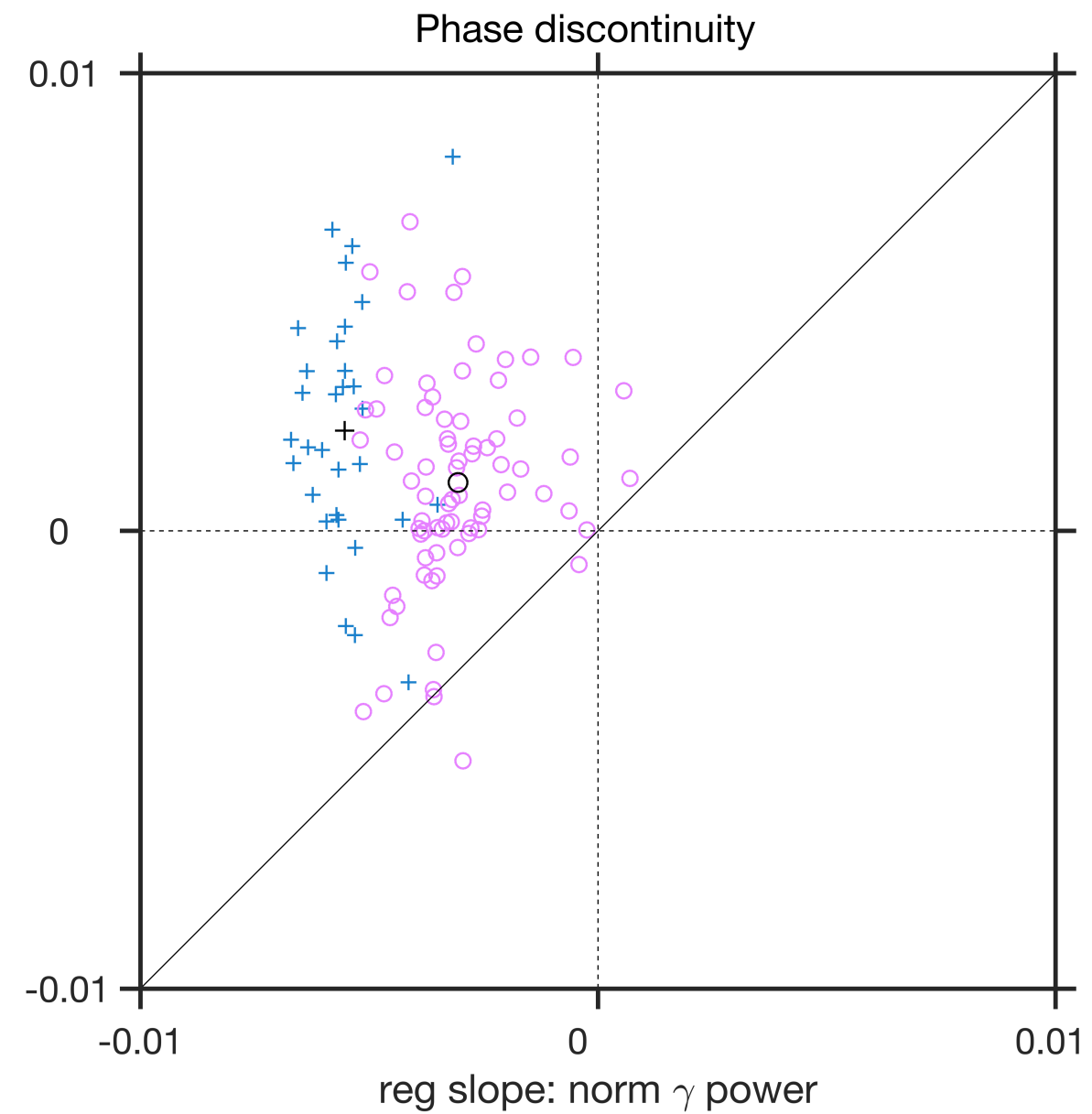

Supplement: S2 Fig — Slope of regression with (A) annulus width, (B) orientation discontinuity (O-I)°, and (C) phase discontinuity (O-I)ϕ° (Δvalue/degree of visual angle) of normalized gamma power versus normalized firing rate. The format of this figure is the same as Figs 1E, 3E, and 4E. The slope of regression for gamma was consistently negative across sites in both monkeys for every type of stimulus discontinuity. The corresponding effects for firing rates were more heterogenous. Therefore, to compare the sensitivity of gamma and firing rates to different stimulus discontinuities, we used the magnitude of slope of regression (Figs 1E, 3E, and 4E). We found no relationship between the slopes for gamma and firing rates (linear regression of slopes for gamma on slopes for firing rates: spatial discontinuity in (A) R2 = 0.002, p = 0.78 in M1, R2 = 0.16, p = 0.01 in M2; orientation discontinuity in (B) R2 = 0.003, p = 0.76 in M1, R2 = 0.03, p = 0.22 in M2; and phase discontinuity in (C) R2 = 0.000, p = 0.97 in M1, R2 = 0.02, p = 0.27 in M2). Figure data are located at https://doi.org/10.5281/zenodo.6523772. M1, Monkey 1; M2, Monkey 2. (PDF) [file pbio.3001666.s002.pdf]

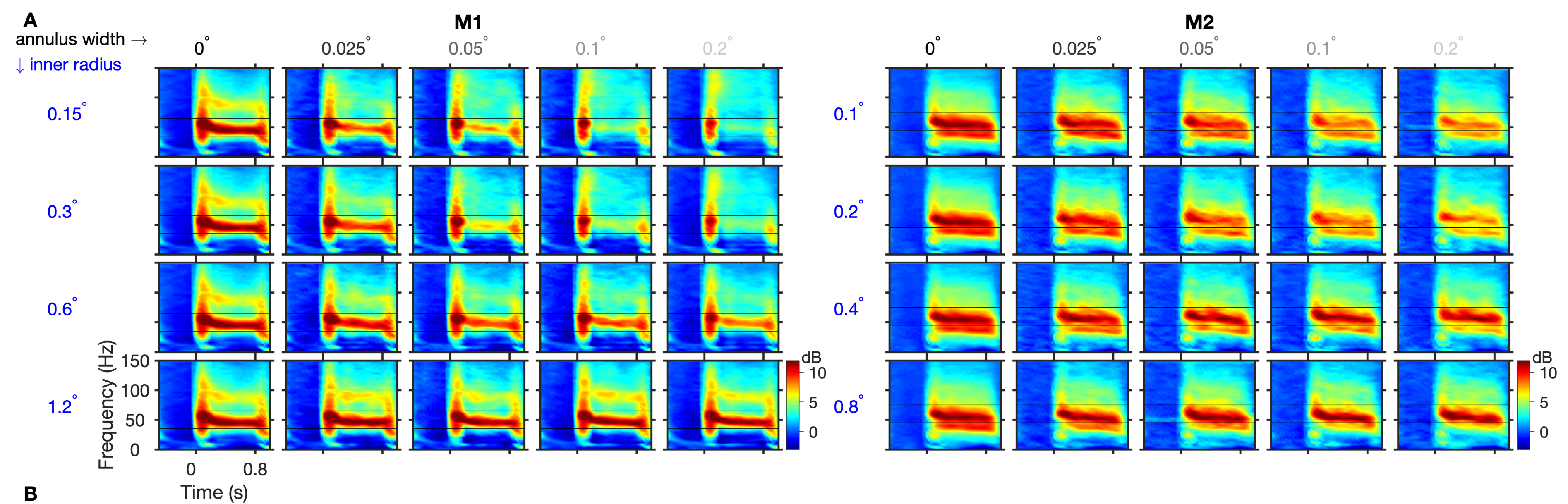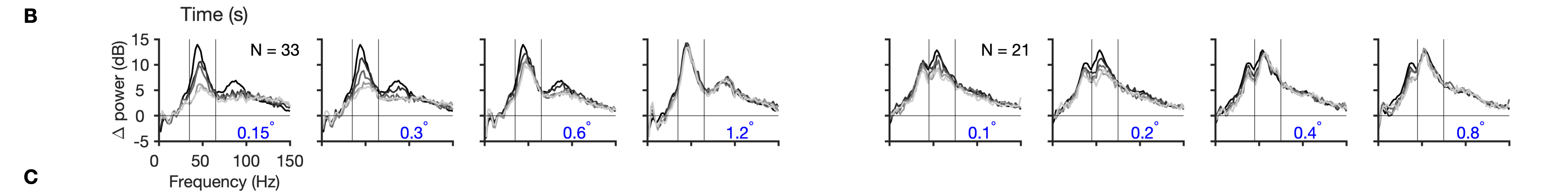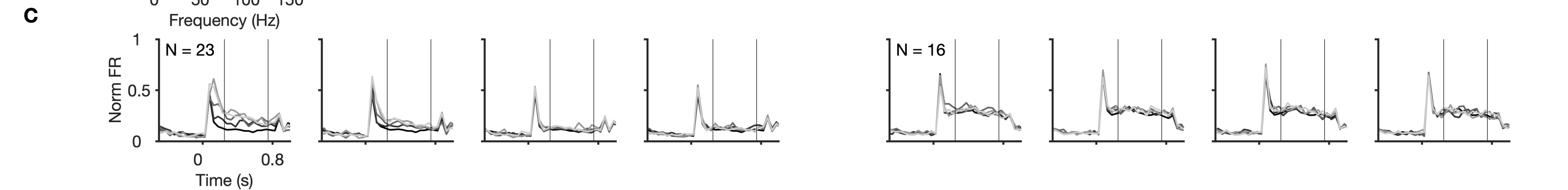

Supplement: S3 Fig — (A) Trial-averaged time-frequency difference spectra for the population in M1 (left column) and M2 (right column), induced by stimuli with annular discontinuity at different inner radii (inner radius and annulus width are indicated on the left and top, respectively) and (B) the corresponding mean change in power from baseline to stimulus period. (C) Mean normalized firing rate averaged across selected spiking center electrodes and sessions. Figure data are located at https://doi.org/10.5281/zenodo.6523772. M1, Monkey 1; M2, Monkey 2. (PDF) [file pbio.3001666.s003.pdf]
