# Supplementary material for: Gamma oscillations in primate primary visual cortex are severely attenuated by small stimulus discontinuities
Source: PLoS Biol. 2022 Jun 14;20(6):e3001666. doi: 10.1371/journal.pbio.3001666 (PMC9197048; doi:10.1371/journal.pbio.3001666)
Supplement: Attachment — Submitted filename: ResponseToReviewers.docx [file pbio.3001666.s004.docx]

**RESPONSE TO REVIEWERS**

Editor’s suggestion

In addition, we would like to suggest a title that is more informative and appealing to a broad readership: “Gamma oscillations in primary visual cortex are a signature of excitation-inhibition balance and attenuated by small stimulus discontinuities.”

*Thank you for this suggestion. This is indeed more informative and appealing to a broad readership. Our only concern is that since we do not explicitly measure the excitation-inhibition balance experimentally, this title may appear a bit speculative. We therefore propose the following:*

*Gamma oscillations in primate primary visual cortex are severely attenuated by small stimulus discontinuities and could be a signature of excitation-inhibition balance.*

*This title has the same information as the one proposed by the editor but is more conservative and experimental findings are given more importance. We hope this is all right.*

Reviewers' comments

Rev. 1:

    The authors recorded from V1 neurons/locations in passively viewing monkeys, using chronically implanted Utah arrays. They varied stimulus discontinuities parametrically along 4 feature dimensions: annular cut discontinuity, orientation discontinuity, phase discontinuity and contrast discontinuity to study the effects of discontinuities on LFP (high/fast) gamma power, and on spiking activity.  They report that for all discontinuities tested gamma power is reduced rapidly, even for small discontinuities, and the changes in gamma power are more pronounced than changes in spiking activity. They build a computational model to explain these effects and report that small changes in excitatory weights (although changes in other weights also affected oscillatory behaviour) in the model result in radically changes in oscillatory behaviour, with only limited effects on overall firing rates. The authors hence argue that changes in gamma power in V1 with stimulus discontinuities is a reflection of E-I balance in the network.

This is an interesting extension of previous work and adds to an important discussion/controversy in the field. While the authors set the framework at least partly in light of the 'binding by synchrony', debate, they do not mention it in the discussion? Maybe this is because the stimuli used are perceptually 'unbound', so they may not provide the ultimate test?

*It is true that the framework for this study is partly based on the ‘binding by synchrony’ debate. However, it does not test that directly. The stimuli are perceptually ‘unbound’ as noted here, and there is no quantitative measure for the perceptual discontinuity for each stimulus or any related behavioral report in the experiment. Therefore, we have avoided discussing the results in the light of the ’binding by synchrony’ hypothesis. However, this is a very valid suggestion and we have now briefly mentioned this point in the discussion (lines 516-518).*

Minor:
    *       line 59: full stop missing
*This has been fixed now.*

*       The authors might consider referencing Bartolo et al.,2011, EJN, who reported related findings.
*We thank reviewer #1 for suggesting this reference! We have now added this at multiple locations in the manuscript (lines 433, 469).*

*       line 315: 'in both the monkeys ..' change to 'in both monkeys'
*We have changed this.*

*       p value reporting: is it meaningful to report exact p-values that are <0.001? It makes more sense to report t-values, z-values, F-values and effect sizes.
*We have added z-values and t-values to make the statistics more informative, as suggested here.*

Rev. 2:

In this work Shirhatti et al. record from macaque primary visual cortex, quantifying changes in spiking and LFP gamma activity to grating stimuli with center-surround discontinuities. The authors show that specifically LFP gamma activity is strongly reduced with progressive stimulus discontinuities across several feature domains. These results are a critical and timely test of growing evidence to suggest visual gamma oscillations are highly sensitive to the structure of visual inputs, which is of functional significance. The authors interpret their findings within the context of excitatory-inhibitory dynamics with visual circuits, providing a computational model based on this interpretation which recapitulates several experimental observations. Overall, these findings are novel and important for understanding the mechanisms of gamma and its function. Below I note several areas where the authors findings require additional discussion.

Main comments:

-Introduction, paragraph (line 53). The authors contrast two alternative accounts for considering the effects of stimulus discontinuities on gamma. However, I think this framing should be reconsidered. Hermes et al. propose a forward encoding model of how stimulus features can be predictive of the observed strength in gamma responses. It is not a biophysical model, and is therefore compatible with several physiological implementations. It is therefore not incompatible with the next mentioned E-I balance models, which focus on circuit mechanisms and not stimulus encoding models. Given that both accounts predict reduced gamma as stimulus discontinuity increases, it seems more accurate to frame these approaches as describing the same putative phenomena at different levels, for which the current study provides critical data.

*This is a good suggestion, and we have now framed the introduction slightly differently in line with it. We have specifically mentioned that the model described in Hermes et al would also predict a drop in gamma amplitude with stimulus discontinuities (lines 56-62). We also state explicitly that since this is an encoding model agnostic to the circuit mechanisms, the relationship between gamma power and discontinuity magnitude could be non-linear. Finally, we have mentioned in the discussion that our data and E-I model confirm well with the predictions from the image-computable model (lines 440-445).*

-Related to the above point, it would therefore be very compelling if in addition to the biophysical model presented, the authors also employed the Hermes et al. stimulus OV model on their stimuli/data. However, I appreciate that this might be beyond the scope of the manuscript.

*This is somewhat beyond the scope of this manuscript, but certainly a good suggestion for future consideration. We briefly discuss this in the Discussion as well.*

-Accurate receptive field (RF) mapping is critical to the design and interpretation of the authors findings. It therefore seems essential that the RF mapping data be presented as an initial figure. The authors should present RF stimulus/response maps with associated RF fitting for both monkeys. Secondly, in comparing spiking and LFP data, is there a difference in the measurement RF that must be considered (e.g. effectively larger RFs for LFP signals)?

*This is a very valid point about requiring careful and accurate characterization of the receptive fields for this study. We have indeed worked on this rigorously and the related details are described in another publication from our lab, which has been included as a reference (17) (Dubey A, Ray S. Cortical Electrocorticogram (ECoG) Is a Local Signal. J Neurosci. 2019 May 29;39(22):4299–311. Monkeys 3 and 4 in that paper correspond to M1 and M2 respectively in this submitted manuscript, albeit from a different electrode grid placed on the other hemifield). That paper describes detailed analyses to characterize the receptive fields computed from LFP and spikes in the monkeys used in this experiment.*

*We have also quantified the relationship between RFs estimated from spikes and LFP power in another cited paper, (54) (Dubey A, Ray S. Spatial spread of local field potential is band-pass in the primary visual cortex. J Neurophysiol. 2016 Aug 3;116(4):1986–99.). The estimates of receptive fields were similar with LFP and spiking responses, with the RF size estimate being only slightly larger with LFP than with spikes.*

As shown below, the results for the two datasets here are largely similar to the two other monkeys shown in the 2016 paper.

We have now included this as a supporting figure S1 to illustrate the receptive fields of the recorded sites in the two monkeys and added a brief description of the mapping procedure.

-For the first experiment, the authors focus on the nature of regression slopes of spiking and gamma power changes across conditions but fail to specifically note that these are in opposing directions. In general, the clear dissociation (often anticorrelation) between spiking and gamma effects seems less noted. However, this divergence in measures seems important for understanding the mechanisms and functional significance of these findings.

-Related to the comment above, many results show a clear anti-correlation or reciprocal relationship between population spiking and gamma results. However, individual scatter plots suggest that this relationship is weak (cf. Fig 1 D vs E). In the discussion, the authors should suggest why this might be. While also noting the implications for why conditions which favor gamma are, on average, associated with low/suppressed spiking.

*We thank the reviewers #2 and #3 for bringing up this important point of investigation and discussion. As suggested by both reviewers, we have shown below the actual values (not just the magnitude) of regression slopes for space, orientation, and spatial phase discontinuities, corresponding to Figure 1E, 3E and 4E of the main paper.*

*These plots provide a better explanation for our choice of using the absolute value of change in firing rates. First, we note that while the changes in gamma power are always negative for all electrodes and conditions, there is more heterogeneity in the firing rates. This is unsurprising, since neurons themselves could be excited or inhibited by stimulation, slight mismatch between the central stimulus size and RF sizes, the exact structure of the excitatory and inhibitory subfields of a neuron and the effect of surround itself which could be suppressive or mildly facilitative (see Angelucci et al (2017)* (1) *for a review). Therefore, simply showing that changes in mean gamma power and mean firing rates (as shown, for example, in Figure 1B-D) is not enough: it is possible that changes in firing rates are also large, but of different signs across population, such that the mean is small. By using the absolute value of the change, as in the current Figure 1E, 3E and 4E, we rule this out.*

*Regarding a possible correlation between the magnitude of changes across electrodes, we found no strong correlation (or anti-correlation) between the spiking and gamma effects in both the monkeys (for example, for the space discontinuity, linear regression of gamma effect on spiking effect: R^2^ = 0.002, p = 0.78 in M1, R^2^ = 0.16, p = 0.01 in M2; similar results for orientation and spatial phase cases as well). This is again unsurprising, since the LFP represents the overall activity of several thousand neurons around the microelectrode, and therefore should not be correlated with the pattern of changes in that one neuron that happened to be better isolated than others during recording.*

*We have now added these as a Supporting figure (S2 Fig) and have discussed the same in the Discussion section and Results.*

-Several experiments show not only a reduction in gamma power with increasing discontinuities, but also an increase in the peak frequency. Is this a consistent phenomenon and how might it fit with the authors E-I mechanism?

*This is a very interesting observation. Indeed, for all the discontinuities, there was an increase in peak frequency (Figure 1B, 3B and 4B) along with a reduction in gamma power, as observed in other stimulus manipulations such as reduction in size (Gieselmann and Thiele, 2008* (2)*) and superposition of an orthogonal grating to make a plaid (Lima et al., 2010* (3)*). We have now included this observation in the Results section.*

*Whether this fits with the E-I mechanism that we have proposed is a difficult question. In our model, effect of a discontinuity is to change the nullclines slightly (by changing the weights) so that the network can generate different behaviors without any substantial change in iI or iE, as shown in Figure 8A-C. The last column in this Figure shows the peak frequency plots. The three traces in Figure 8D have frequencies of 46, 47.5 and 46.5 Hz. So there is indeed an initial increase in peak frequency, consistent with data, but there is also a subsequent reduction in peak frequency with increasing discontinuity.*

*Since the range over which the weights change with discontinuity is arbitrary, we could in principle constrain the model to operate in a range that fits the data. However, we think this is overselling the model. The changes in peak frequency are very small and also inconsistent going from 8A to 8C (unlike the power effects that are really robust). We think a more detailed model is needed, which can perhaps also explain other stimulus manipulations such as a plaid stimulus (perhaps similar to the variance model described above), to explain the peak frequency effects. Given these constraints, we have refrained from discussing the peak frequency shifts in the E-I model here, although we fully agree that this is an interesting observation which can potentially be used to further fine-tune the model.*

-Several studies in primates report no evidence of gamma oscillations in the thalamus (LGN), suggesting a cortical mechanism. It appears the authors interpretation support this view, given the focus on E-I physiology and connectivity within visual cortex. This should be noted in the discussion.

*We thank the reviewer for this suggestion and have noted this in the discussion now (lines 487-490).*

-How do the authors experimental and computational findings relate to the similar modelling work of Heeger and Zemlianova (2020, PNAS)? (which should be cited).

*Heeger and Zemlianova (2020, PNAS* (4)*) have developed a family of dynamical systems with recurrent amplification to implement normalization. Recurrent models from this family recapitulate a number of known experimental observations in V1, including gamma oscillations. In comparison to the lumped model used by us, their model has a more detailed structure (units called as modulator cells) to specifically implement recurrent connections and amplification. Their model uses units with different RF centers, orientation preferences, spatial and temporal phases, and the weights in the system implement tuned normalization. Despite the differences in structural detail their model is similar to our model in certain aspects. Both are like the Inhibition-stabilized network in essence. Similar to a restricted valid regime and E-I drive in our model, gamma oscillations are generated in some models of their family for a restricted set of stimulus conditions, and they depend on the strength of the normalization pool. Some of their models exhibit strongest gamma oscillations for large, high-contrast gratings, but not for other spatial patterns or low-contrasts. In their family of models, discontinuous stimuli, like the ones used in our experiments, would shift the responses of their principal cells and modulator cells due to tuned normalization-based system weights. This would effectively change the recurrent gains (which depend on the activity of the modulator cells) in the network, similar to the change of weights shown in our model. Considering the similarities, their family of models should potentially be able to replicate the experimental findings presented here with discontinuous stimuli, although that needs to be tested to confirm. We thank the reviewer for pointing out this crucial reference and have now cited it in the manuscript.*

-Overall, recent experimental and theoretical work has repeatedly linked center-surround effects in V1 to the genesis of strong gamma oscillations. Importantly, for gamma, it is congruency between center-surround stimulus features which is optimal. Alternatively put, classic spiking surround-suppression conditions (which decreases spiking) enhance gamma. Given these tight links to a larger literature, and recent arguments about such a mechanism reflecting a ‘predictive’ function of gamma, it would be important for the authors to directly engage how their findings/interpretation relate to this literature. Specifically, how does the authors E-I account align with the known physiology of surround-suppression, such that it’s consistent with spike reduction and gamma enhancement. While I note the authors do engage with these ideas, the implication is often just that the E-I balance will be changed- which verges on being trivial (true for any input/event in cortex). Given the consistent parametric effects on gamma observed, should their not be a more specific interpretation for how the E-I balance is specifically changing under these conditions?

*We thank the reviewer for this interesting idea. Indeed, several studies have discussed about the potential role of gamma in predictive coding, which could in turn be related to how centre-surround computations are implemented. We now discuss this idea and some of these papers. We don’t think it is straightforward to test the predictive coding hypothesis with these data since it is unclear how the predictability varies with the magnitude of discontinuity.*

Minor comments:

-While experimental conditions are color matched via stimuli shown in figures, legends for lower panels where color line plots are used would benefit the reader.

*We have now added legends for the lower panels in Fig 1, Fig 3 and Fig 4 to make it easier to read the plots.*

Rev. 3:

This study measures two kinds of neural signals, gamma oscillations and action potentials, from monkey primary visual cortex using microelectrode arrays. The main question addressed is how discontinuities in visual stimuli affect the two signals. Several discontinuities are introduced between the center and surrounding regions of an image patch, approximately centered over the receptive field of the mircroelectrodes. The discontinuities include a zero-contrast annulus, an orientation difference, a phase difference, and a contrast difference. In all 4 cases, the extent of the discontinuity is systematically varied (width of the annulus, orientation difference, phase difference, contrast difference).  The main finding is that all discontinuities tend to result in a large decrease in the power of gamma oscillations and a smaller increase in the spike rates. The motivation for the study is not entirely clear, but generally it is suspected that image discontinuities are likely to impact gamma oscillations, and that this might provide some clues into the functional and biological significance of the oscillations. Finally, a widely used neural model comprised of interconnected excitatory and inhibitory units (“E/I”) is implemented. The model does not directly allow for stimulus properties to be input, but discontinuities in the stimuli are simulated by varying some model parameters, which in turn shows that small perturbations in model parameters can result in large changes in oscillatory behavior of the model, with little change in mean signal level, interpreted as supporting the empirical observations. The main contribution of the paper is the empirical findings from the systematic manipulation of multiple types of stimulus discontinuities while measuring LFP and spiking. The fact that relatively small image discontinuities have large effects on the oscillations is likely to be important for understanding the significance (and perhaps biological origin) of the signals, though no strong conclusions are drawn about this. Nonetheless, the results are clear and likely to be of interest to the field.

Major comments:

(1) I see no major problems with the data analysis or experimental methods. But surprisingly, what seems to me to be the biggest and most consistent effect in the dataset is barely commented on at all: the fact that discontinuities in the stimuli cause firing rates to increase and oscillations to decrease. The fact that the two signals are affected in opposite ways might have some implications for neural coding, and it is odd that the authors are primarily only interested in comparing the absolute value of the two signals, rather than the fact they change in opposite directions as a function of their stimulus manipulations.

*This is an important point and we have included an explanation for this in the responses to reviewer #2 (lines 135-160 above). We have now added a supporting figure (S2) to show the relation between changes in gamma and firing rates due to discontinuities and explained the choice of absolute value of the regression slopes to compare their sensitivities. That said, there are indeed many instances where gamma and firing rates change in opposite ways due to stimulus manipulations used in this study. We have now explicitly stated and discussed this opposite trend in gamma and spiking effects in the Discussion.*

(2) The firing rates are discussed as though they are only slightly modified by the discontinuities, compared to the enormous effects on oscillations. Yet the firing rate effects look robust as well. Clearly the effects on oscillations are bigger, but the firing rate effects are not small: they look to be about half the size of the effect on oscillations. Without a theory to help us interpret the meaning of these effects, it is difficult to say whether one, both, or neither, are big enough to matter. In any case, the difference between the two is somewhat overstated in places.

*It is true that the changes in firing rates are significantly large and robust for certain steps of stimulus discontinuities, despite being smaller than the effect on gamma oscillations. We have now discussed this and related points in more detail in the ‘Discussion’ section (lines 457-473). The difference in the effects for spiking and gamma responses could be due to differences in their tuning properties and different scales of the neuronal network influencing them.*

(3) It looks like in the model, the effects on firing rates are smaller and go in the opposite direction compared to the data (if I am understanding the results correctly). This does not take away from the fact that the model shows much larger sensitivity of the oscillations than of the mean with respect to model parameters. But it would seem to warrant mention.
*We have specifically mentioned this point in the manuscript now (lines 399-400).*

Overall, this is a very well-done empirical study.

*We thank the reviewer for positive and encouraging comments about our work.*

*REFERENCES*

1.	Angelucci A, Bijanzadeh M, Nurminen L, Federer F, Merlin S, Bressloff PC. Circuits and Mechanisms for Surround Modulation in Visual Cortex. Annu Rev Neurosci. 2017;40(1):425–51.

2.	Gieselmann MA, Thiele A. Comparison of spatial integration and surround suppression characteristics in spiking activity and the local field potential in macaque V1. Eur J Neurosci. 2008 Aug;28(3):447–59.

3.	Lima B, Singer W, Chen NH, Neuenschwander S. Synchronization Dynamics in Response to Plaid Stimuli in Monkey V1. Cereb Cortex N Y NY. 2010 Jul;20(7):1556–73.

4.	Heeger DJ, Zemlianova KO. A recurrent circuit implements normalization, simulating the dynamics of V1 activity. Proc Natl Acad Sci. 2020 Sep 8;117(36):22494–505.
